# Accelerating First Principles Calculation of Multi-Component Alloy Steady-State Structure and Elastic Properties in Full Component Space

**DOI:** 10.3390/ma16186226

**Published:** 2023-09-15

**Authors:** Zhixuan Yao, Yan Zhang, Yong Liu, Mingwei Li, Tianyi Han, Zhonghong Lai, Nan Qu, Jingchuan Zhu, Boyuan Yu

**Affiliations:** 1 School of Materials Science and Engineering, Harbin Institute of Technology, Harbin 150001, China; 2 National Key Lab for Precision Heat Processing of Metal, Harbin Institute of Technology, Harbin 150001, China; 3 Center of Analysis, Measurement and Computing, Harbin Institute of Technology, Harbin 150001, China; 4 School of Safety Engineering, China University of Mining and Technology, Xuzhou 221116, China

**Keywords:** multi-component alloy, first principles calculation, machine learning, elastic property

## Abstract

The FeNiCrAlCoCuTi alloy system has great advantages in mechanical properties such as high hardness and toughness. It has high performance potential and research value and the key in research is designing alloy compositions with target properties. The traditional method, experimental analysis, is highly inefficient to properly exploit the intrinsic relationship between material characteristics and properties for multi-component alloys, especially in investigating the whole composition space. In this work, we present a research way that uses first principles calculation to obtain the properties of multi-component alloys and uses machine learning to accelerate the research. The FeNiCrAlCoCuTi alloy system with its elastic properties is used as an example to demonstrate this process. We specifically design models for each output, all of which have RMSE values of less than 1.1, and confirm their effectiveness through experimental data in the literature, showing that the relative error is below 5%. Additionally, we perform an interpretable analysis on the models, exposing the underlying relationship between input features and output. By means of spatial transformation, we achieve the prediction of the full-component spatial performance from binary to multiple components. Taking the FeNiCrAlM (M = Co, Cu, Ti) quinary alloy system as an example, we design a single-phase BCC structure composed of an Fe_0.23_Cr_0.23_Al_0.23_Ni_0.03_Cu_0.28_ alloy with a Young’s modulus of 273.10 GPa, as well as a single-phase BCC structure composed of an Fe_0.01_Cr_0.01_Al_0.01_Ni_0.44_Co_0.53_ alloy with a shear modulus of 103.6 GPa. Through this research way, we use machine learning to accelerate the calculation, which greatly shortens research time and costs. This work overcomes the drawbacks of traditional experiments and directly obtains element compositions and composition intervals with excellent performance.

## 1. Introduction

The conventional compositional design strategy of alloys always begins with only one or two principal elements and followed by the additional elements [1]. Recently, multi-component alloys (MCAs) and high entropy alloys (HEAs) [2,3] were introduced with mixed multiple elements in equimolar or near-equimolar compositions, as a novel type of alloy design approach. In the dilemma of limited material types, multi-component alloys have gradually become a research hotspot. The increase in the variety of elements and the expansion of the composition range have led to a rapid increase in the potential variety and performance of multi-component alloys. Benefitting from their unique features including a high entropy effect, a severe lattice distortion effect, a hysteretic diffusion effect and a cocktail effect [4,5], as well as their unusual properties, such as outstanding mechanical properties [6,7,8,9], a high hardness and wear resistance [10,11] and an exceptional high-temperature strength [12,13], they receive intensive attention. So far, MCAs provide opportunities for material researchers to design new materials with desired properties, based on the wide compositional space in which MCAs can be formed [14]. The increasing number of MCAs also expands the potential candidates for next-generation materials in a wide range of fields than traditional alloys. The FeNiCrAlCoCuTi alloy system, a typical multi-element alloy system, has great advantages in mechanical properties [15,16,17]. It has a high performance potential and research value and the key in research is designing alloy compositions with target properties.

Changing the composition and range of elements to achieve better performance is a common approach in the study of multi-component alloys. Heretofore, the traditional development process of new materials still adopts the trial-and-error method, and the experimental steps are cumbersome. It takes 15–25 years or even longer cycles from research and development to application. The long cycle and high cost limitations make it difficult to accelerate the development of materials [18]. In addition, more and more techniques are used for material characterization, and the corresponding data volumes and dimensions are becoming more and more complex. Traditional methods of experimental analysis that rely on manual methods sometimes fail to properly exploit the intrinsic relationship between material characteristics and properties [19]. At the same time, investigating this large compositional space of alloy designs using the conventional trial-and-error method is highly inefficient. As an alternative, the rise of computational simulation has become a powerful tool for discovering new materials. High-precision calculation methods such as the DFT-based first principles calculation [20], the Monte Carlo simulation [21], the molecular dynamics simulation [22], the phase field method [23] and the finite element analysis [24] are widely used in new materials discovery. Especially for the first principles calculation, material researchers have previously used this approach to predict the phase stability of alloys or to analyze the strengthening mechanism of alloys [25,26,27,28,29,30,31]. However, it is often applied only to specific systems; the computation of complex systems and the development of theoretical methods that do not meet the requirements for quantitative characterization of material properties is unaffordable [32]. Therefore, those who design MCAs need an approach that will allow them to quickly and efficiently search for an MCA with desired properties. It is necessary to introduce new methods into the research line to accelerate the calculations.

Machine learning (ML), a data-driven approach, has been employed to predict the mechanical properties, such as yield strength, hardness, elastic modulus and critical resolved shear stress of MCAs as well as several other alloys [33,34,35,36]. Furthermore, material researchers have found that this can overcome the limitations of the above-mentioned approaches [37,38,39]. The aforementioned studies successfully predicted properties using ML, but most of them did not design alloys or conduct experimental verifications of the designed alloys. ML balances the computing cost and calculation accuracy well in the development of novel materials. The advance in applying ML to first principles calculation provides new opportunities for balancing calculating accuracy and cost [40,41,42]. This is because the ML workflow bypasses the computationally costly step of solving the Schrödinger equation [43]. Compared with solving the Schrödinger equation, the computational speed of the ML process is faster by several orders of magnitude.

**Figure 1:**
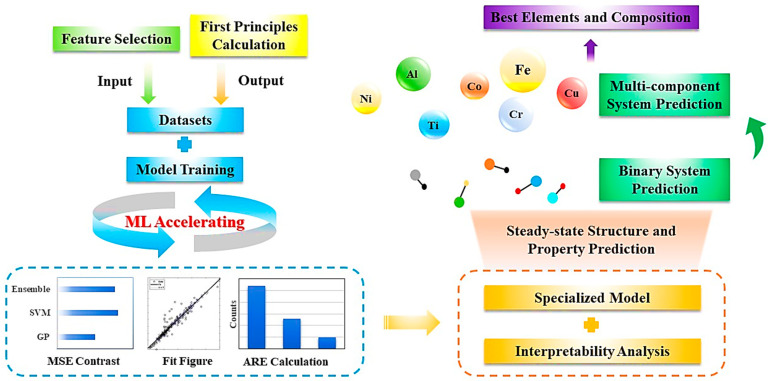
A research way of first principles calculation and machine learning for steady-state structure and property prediction of multi-component alloys based on their composition.

In this work, we present a material research way that uses first principles calculation to obtain structure and properties of multi-component alloys and uses machine learning to accelerate the process, as shown in Figure 1. The FeNiCrAlCoCuTi alloy system with its structure and elastic properties is used as an example to demonstrate this process. Using this research way, we systematically study the relationship between the composition, structure and properties. The typical research way consists of three necessary steps. First, first principles calculation is used to obtain the system energy, cell volume, Young’s modulus (E), bulk modulus (B) and shear modulus (G) of the unit and binary system in the MCA, resulting in a total of five properties as ML outputs. Then we use feature engineering to simplify the inputs in order to simplify the models, from which the composition is transformed into descriptors including phase parameters and mechanical parameters. Special descriptors are designed for each output by calculating the mean squared error and the importance, which are then used as inputs for ML models. Thus, the construction of composition–structure and composition–property datasets are completed. Second, three algorithms are selected to train ML models, and the best model is determined through the mean squared error, relative error and regression figure of the model, which ensures the accuracy of the first principles calculation under the condition of saving calculation cost. Subsequently, we perform an interpretability analysis on the models, intuitively reflecting the contribution of each input feature to the outputs. The models specially designed for each output show good prediction effects. This point is verified through validation via comparing experimental data in other studies and our predicted results. Third, through spatial transformation, we realize the prediction of the steady-state structure and elastic properties of the full component space from the binary system to the multivariate system. We also directly obtain the composition corresponding to the best performance. Taking the FeNiCrAlM (M = Co, Cu, Ti) quinary alloy system as an example, we design single-phase alloys with higher moduli.

Our material research way overcomes the drawbacks of traditional experiments and simulation calculations, to quickly and effectively search for multi-component alloys with the required properties, and directly obtain element compositions and composition intervals with excellent performance. When designing new materials, the characteristics and advantages of multiple alloys can be better utilized, and new materials with better or similar mechanical properties can be designed in less time. This gives effective guidance for performance prediction and composition. It can also accelerate the design process of new MCAs.

## 2. Modeling and Dataset

### 2.1. First Principles Calculations

Machine learning was carried out on a dataset consisting of input and output parts, and involved four main steps: (I) data collection and preprocessing, (II) feature engineering, (III) model selection and training and (IV) model evaluation and optimization. Data can be collected from the published literature or reference books, obtained from simulations and calculations, obtained in laboratory experiments through high-throughput experimental techniques, citing data from high-quality databases, or a combination of the above methods. In materials science, BCC, FCC and HCP have a common tetrahedral unit which contains information about the atomic positions and interactions. Modeling the disordered phase structure of high-entropy alloys poses a significant challenge. But when focusing on several atoms at the cluster scale, they exhibit similar characteristics of tetrahedral units which results in common structures and properties. In the case of the increase in the number and type of atoms in a material system, the prediction of the structure and properties of the multi-component material become possible because of the interaction between various atoms and the corresponding tetrahedral structural units divided in the multi-component system. Thus, we used first principles calculation to obtain the system energy, cell volume, Young’s modulus, bulk modulus and shear modulus of the unit and binary system as the outputs of the machine learning models. Thereunto, the system energy and cell volume were used to set the composition–structure dataset, while the Young’s modulus, bulk modulus and shear modulus were used to set the composition–property dataset. It is hoped that these data can be used to achieve a leap towards a multi-component system. It is worth noting that first principles calculations provide the intrinsic structure and properties of alloys without considering the influence of temperature.

A total of 21 different unit cell models were established for three different structures, which are BCC, FCC and HCP. In the case of binary systems, 336 models were created with different compositions and structures, including perfect A2B2, A1B3 and defective A1B2 structures. In order to reduce the computational time, the model size should be simplified as much as possible. Shell scripts were also used in the calculation process to replace the corresponding elements of the A and B positions, to create batches of crystal structures of binary systems and to submit tasks, from which we achieved high-throughput first principles calculations.

**Figure 2:**
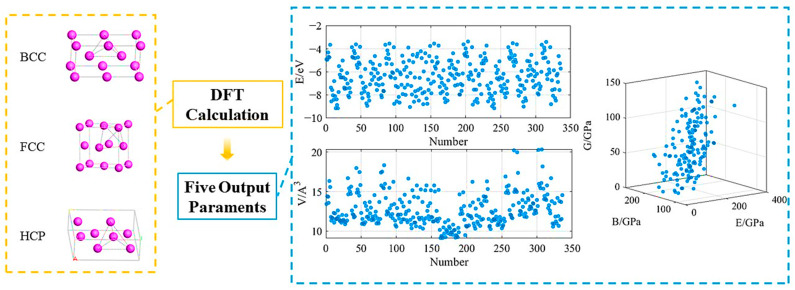
Distribution of machine learning output values of the unit and the binary system of the FeNiCrAlCoTiCu alloy system, calculated using first principles calculation.

We initiated calculations from the binary phase of the FeNiCrAlCoTiCu system and employed vacancy construction unit systems to design composition gradients with an interval of 0.25. Considering the significant influence of the phase composition on the material properties, calculations were performed for three types of phase structures: BCC, FCC and HCP. Consequently, a total of 336 sets of structural property data and 152 sets of elastic property data were obtained, and their distributions are shown on the right side of Figure 2.

Figure 2 shows that five output values are evenly distributed in the graph as the types and structures of the elements vary. Therefore, it is necessary to organize and analyze the data systematically. We came to the following conclusions. In the cell system, the variation in the energy of the monatomic unit with the change in atomic species exhibits a similar trend in the three structures of BCC, FCC and HCP. There are two peaks, located at the positions of the elements Cr and Ti, respectively. The pure elements Fe and Cr exhibit the most stable BCC structure, while Ni, Al and Cu have the most stable FCC structure. Co and Ti exhibit the most stable HCP structure, which is consistent with the actual situation. In a binary system, the system energy is primarily concentrated in the range of 5–10 eV and exhibits the same changing trend with variations in the atomic species of element B. It is also found that positions A and B possess certain equivalences. When element A is Fe or Cr, the system has a lower energy, and phases containing these two elements are relatively easier to form. When element A is Al, Cu or Ni, the system has a slightly higher energy, and the formation of phases containing these three elements requires more external energy input. The energy of the BCC and FCC structures is generally lower than that of HCP, making these system structures relatively more stable.

The overall trend of the cell volume change in the elemental crystal cells is relatively consistent, with peaks observed in Al and Ti. In binary systems, the trends of the crystal cell volume for the A1B1, A1B2 and A1 components are consistent with the trends observed in the single component system, showing an increase when the B element is Al or Ti, with A1B1 and A1 showing a more significant increase. Among these components, Al exhibits a much higher increase compared to the others, while A1B3 and A2B2 show relatively small overall changes. In the HCP structure, A1B1 exhibits a significant decrease when the substitution elements at positions A and B are the same.

In the elastic constants of unit cell systems, BCC Al, Ni and Ti; FCC Fe and Cr; and HCP Cr have negative values for their Young’s moduli and shear moduli, indicating unstable structures. The changes in the trends of the Young’s moduli and shear moduli are basically consistent. When calculating binary elastic constants, the influence of different initial magnetic moments set by spin polarization on the calculation results is significant, mainly based on the bonding situation. The trends in the Young’s moduli and shear moduli for the three different structured components are consistent.

The first principles calculations were performed with the Vienna Ab-initio Simulation Package (VASP). The generalized gradient approximation (GGA) was employed in all calculations. The pseudopotential method using ultra-soft pseudopotentials in the plane wave basis set was employed to describe the interaction between ions and electrons. A plane wave basis with a cut-off energy of 500 eV and 9 × 9 × 9 k-sampling in the Brillouin zone were used for all calculations. The electronic convergence accuracy was 10^−8^ eV. The first principles calculation strategy we adopted starts from the elemental composition and structure of multi-component alloy systems with generality and high throughput characteristics, making it suitable for various types of multi-component alloys. Through the aforementioned process, we successfully completed the data acquisition for the outputs of the composition–structure and the composition–property datasets.

### 2.2. Feature Engineering

**Figure 3:**
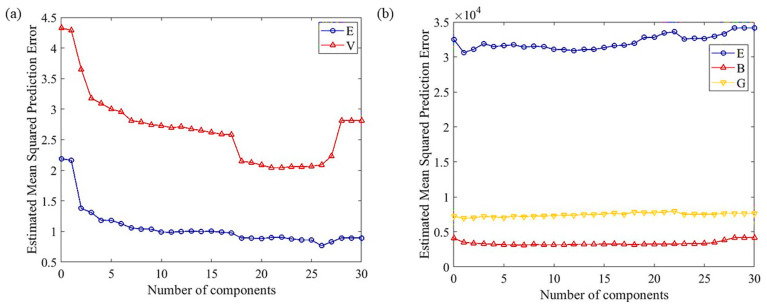

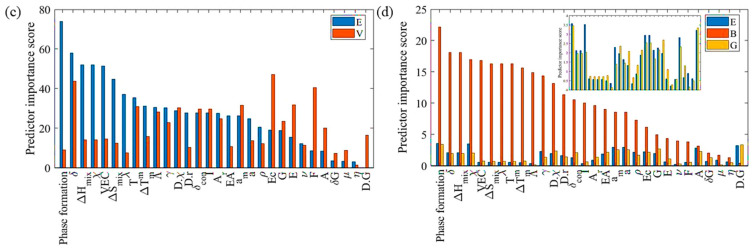
The mean square error of (**a**) system energy, cell volume and (**b**) Young’s modulus, bulk modulus and shear modulus calculated as a function of the number of input features; the importance of each feature for predicting (**c**) structure and (**d**) elastic property outputs.

Although we aim to study the relationship between composition, structure and properties in multi-component alloys, the composition cannot serve as a direct input for machine learning models. The immense compositional space will inevitably increase the complexity of the machine learning model and decrease its accuracy. Selecting appropriate descriptors related to the composition can effectively solve this problem. To reduce the complexity of the model, the number of descriptors can be limited to a certain value without compromising the performance of the model [44]. In fact, some reports claim that the model degrades the results when there are too many variables because the redundant feature variables interfere with the ML model [45]. Thus, it is essential to down-select the most significant and relevant features to construct ML models. We selected a series of descriptors including phase parameters and mechanical parameters from the literature, as listed in Supplementary Tables S1 and S2. In addition to the parameters listed in Supplementary Table S1, the phase formation parameter was also included. We collected values for the atomic radius, valence electron density and melting point for lattice parameters and elastic constants calculated through first principles calculations for binary systems of seven elements in diverse alloy systems. We then used the formulas listed in Supplementary Tables S1 and S2 to calculate the values of all descriptor parameters for each binary system. Partial least squares regression (PLS) was used to calculate the effect of the number of features on the models’ mean square error (MSE), as shown in Figure 3a,b. The MSE calculation method was specified as a resubstitution, with a Monte Carlo repetition of 1 in the cross-validation. And a 10 folds cross-validation was used to analyze the above features.

In Figure 3a, the MSE of the system energy and cell volume decreases rapidly with an increasing number of inputs, followed by a slower decrease. The MSE of the cell volume further reduces significantly when the number of inputs reaches 15–20, and then stabilizes for a period before increasing again. In Figure 3b, the MSE of the modulus E decreases initially and then slightly increases with an increasing number of inputs, while remaining at a relatively high level. The moduli B and G on the other hand, show little variation with the number of inputs. Taking into consideration the impact of the number of inputs on the MSE of all outputs and the risk of overfitting associated with excessive inputs, we ultimately selected six as the number of input features for the subsequent model training.

After analyzing and determining the optimal number of inputs, further consideration is given to selecting which six descriptors can achieve the highest accuracy of the model. In this work, we calculated the importance of thirty features in predicting the system energy, cell volume and elastic properties. Feature importance is determined by assigning a value to input features based on how relevant they are in predicting a target property [44], which is shown in Figure 3c,d. Regarding the energy of the system, the most prominent features are the phase formation, δ, ΔH_mix_, χ, VEC and ΔS_mix_, all of which relate to phase parameters. This is consistent with the pattern formed as described in reference [46]. Regarding the cell volume, the top six features in order are the phase formation, δ, a_m_, E_c_, E and F, with two of them being phase parameters. Among the elastic constants, the importance of the bulk modulus stands out compared to the others. Therefore, the bulk modulus was given priority in the sorting. The upper right corner of Figure 3d shows an enlarged view of the importance scores of the Young’s modulus and shear modulus. For the bulk modulus, the top-ranked features are the phase formation, δ, ΔH_mix_, χ, VEC and ΔS_mix_, all of which are phase parameters. Regarding the Young’s modulus, it primarily depends on the phase formation, χ, a_m_, a, A and D.G., whereby two of these are phase parameters. In regards to the shear modulus, the main factors include the phase formation, D.χ, a_m_, a, G and D.G, wherein two of these are phase parameters.

**Figure 4:**
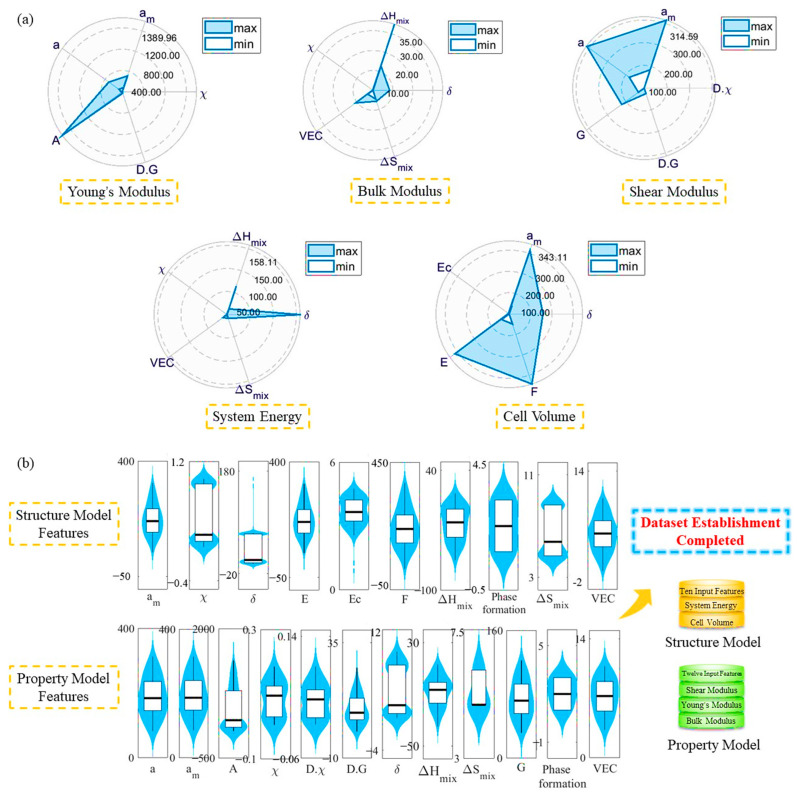
(**a**) The input features were selected for each output by importance and are organized for use, and (**b**) similar features are combined to simplify calculations and achieve the establishment of the composition–structure and composition–property datasets.

We performed data sorting on each output without the phase formation for further analysis, as shown in Figure 4a. It is evident that the feature values of each feature are controlled within a certain range, which effectively ensures the accuracy of the machine learning models. In order to simplify the calculations, we further merged identical features to construct the composition–structure and composition–property datasets. Through the aforementioned feature engineering, we simplified the input and in turn simplified the model.

### 2.3. Machine Learning Design

To predict the steady-state structure and elastic properties of the full component space from the binary system to the multivariate system, we chose three commonly used algorithms, including support vector machine (SVM), ensemble regression (ER) and Gaussian processes (GP), to establish ML models describing the relationship between composition, structure and elastic properties. Support vector machine is a linear classifier that finds the maximum margin in the feature space [47]. Its core idea is to achieve data separation by constructing a separating hyperplane. Ensemble regression does not rely on a single learner but constructs multiple learners with a certain strategy and combines them together to accomplish learning tasks more effectively than individual learners [48]. According to the relationship between individual learners, it can be divided into three categories, including bagging, boosting and stacking. Gaussian processes is a non-parametric model that performs regression analysis on data [49]. The significant difference from other regression algorithms is that it obtains the function distribution rather than the output value corresponding to the input, and is suitable for modeling nonlinear systems. The advantage of Gaussian processes is that it can flexibly adjust the complexity of the model according to the training data, and it can also guarantee the boundedness of the prediction error to a certain extent.

By adjusting the model parameters, the accuracy of a regression can be improved. Underfitting and overfitting are common issues in small datasets during the training process [50,51]. Appropriate parameter design can effectively solve this problem. Therefore, we chose 2 as the polynomial order for the SVM algorithm. We chose 1 as the LearnRate value for the ER algorithm. We chose 0.01 as the regulation for the GP algorithm. You can find more details for the algorithm parameters in Supplementary Table S3; they are reproducible and can guide others in parameter design. We used k-fold cross-validation to cross-validate the machine learning models for each algorithm. We also employed normalization to further enhance the accuracy of the models.

In order to evaluate the effectiveness of a machine learning model and its parameters, we should not only consider the correlation and coincidence between the predicted values and the actual values, but also take into account the overall dispersion and stability, so the correlation coefficient (R), the mean square error (MSE) and the absolute relative error (ARE) were selected to evaluate the effectiveness of the model.

## 3. Results and Discussion

### 3.1. Feature Analysis

Multi-component alloys are diverse in type, and as the number of elements increases, they have a microstructure that is completely different from traditional alloys. Compared to the composition of various intermetallic compounds in traditional alloys, multi-component alloys tend to form simple solid solution structures due to the high entropy effect. The microstructure of multi-component alloys plays a decisive role in their performance, so it is essential to identify the phase composition and microstructure of multi-component alloys in order to study their properties. Figure 4a displays the input features that were specifically selected for the five outputs. Among the five outputs, phase formation represents one of the six descriptors that were ultimately selected. It can be observed from the figure that the phase formation has a significant impact on the output, which is consistent with the findings of other studies [50]. And for both the system energy and bulk modulus, the six descriptors selected are consistent. Among the parameters such as phase formation, δ, ΔH_mix_, χ, VEC, ΔS_mix_, a_m_, a and D.G, several were selected more than once, and they have a greater impact on the model’s prediction performance among the 30 features selected. For predicting the stable structure and elastic constants of the system, the proportion of the mechanical parameters selected is lower than that of the phase parameters. Except for predicting the system energy and bulk modulus, which are both phase parameters, the proportion of the other mechanical parameters is 0.33. Therefore, identifying the equilibrium phase composition corresponding to different types and multi-component alloys is of great significance for performance research.

For a general multi-component system, the Gibbs energy can be expressed by the following Equation (1)

(1) $$G=\bar{G}+R_{g}T\Omega ((\frac{1}{V_{1}}-\sum_{i=2}^{n}(c_{i}-\sum_{j=2}^{n}c_{ij}))ln(1-V_{1}\sum_{i=2}^{n}(c_{i}-\sum_{j=2}^{n}c_{ij}))\;+\sum_{i=2}^{n}{((c}_{i}-\sum_{j=2}^{n}c_{ij})ln({(c}_{i}-\sum_{j=2}^{n}c_{ij})V_{1}))\;+\sum_{i=2}^{n}((\frac{1}{V_{i}}-\sum_{i=2}^{n}c_{ij})ln(1-\sum_{j=2}^{n}c_{ij}V_{i})+\sum_{j=2}^{n}c_{ij}ln(c_{ij}V_{i}))\;-\Omega [\sum_{i=2}^{n}\sum_{j=2}^{n}\frac{\varepsilon_{ij}c_{ij}}{2}+\sum_{i=2}^{n}\varepsilon_{i1}({Zc}_{i}-\sum_{j=2}^{n}c_{ij})+\varepsilon_{11}(\frac{Z}{2\Omega }-Z\sum_{i=2}^{n}c_{i}+\;\sum_{i=2}^{n}\sum_{j=2}^{n}c_{ij}/2)]$$

where $\bar{G}$ (J/mol) represents the Gibbs energy term that is independent of the system’s internal state variables and configurational entropy. Ω (m^3^/mol) denotes the volume of the system, while Z is the number of neighboring atoms. c_ij_ (mol/m^3^) represents the atomic concentration of component i, which is the nearest neighbor to component j. Finally, ε_ij_ (J/mol) is the energy associated with the bond between atoms of components i and j. And the mixed Gibbs free energy can be expressed by the following Equation (2)

(2) $$∆G_{mix}=∆H_{mix}-T∆S_{mix}$$

where ΔH_mix_ (J/mol) is the enthalpy of mixing, ΔS_mix_ (J·mol^−1^·K^−1^) is the entropy of mixing, ΔG_mix_ (J/mol) is the Gibbs free energy of mixing, and T (K) is the temperature. Based on the above formulas, there is a close relationship between the ΔH_mix_ and ΔS_mix_ of the system and its free energy. The system’s volume is closely related to the lattice parameters, corresponding to a_m_ and a. Most of the selected features are based on the mixing rule, including the atomic concentration term, which corresponds well to the free energy formula. The bond energy between atoms greatly affects the electronegativity of the system, corresponding to χ and D.χ. E, F, G and D.G are parameters related to mechanical performance. E_c_ and A themselves are energy terms. Therefore, the Gibbs free energy of multi-component systems can be described by a certain linear combination of the selected descriptors. This description not only exhibits numerical correlations, but also aligns well with thermodynamic expressions, and possesses certain physical significance.

### 3.2. Model Evaluation and Interpretable Analysis

**Figure 5:**
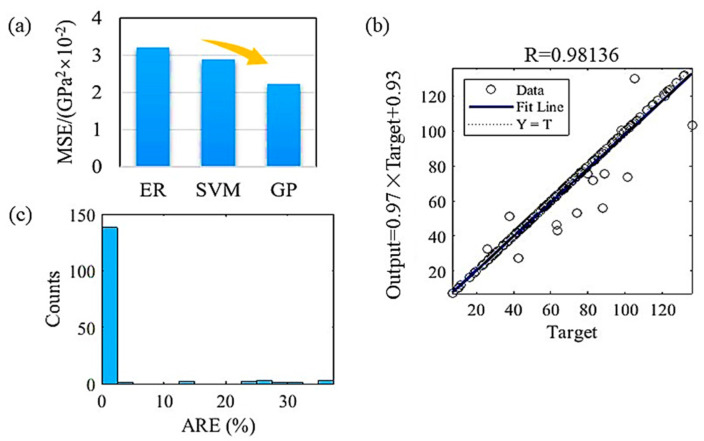
Regression results of the GP model for the shear modulus including (**a**) the MSE, (**b**) the regression figure and (**c**) the ARE.

The superiority of our model can be examined by comparing the accuracy of the model with other possible ML models with various feature sets and model types. However, a direct comparison of the performance with models published in the literature referenced in the above section was not attempted. This is because the purposes and inputs of those models are different from ours. It may not be fair to compare the ML modeling performance of models with different purposes because the performance can vary depending on the target properties. Thus, in our process of establishing ML models, we select three commonly used algorithms and aim to select the most suitable algorithm and corresponding model by evaluating it with metrics including the regression figure, MSE and ARE. Taking the shear modulus as an example, the GP model presents a higher R value, as shown in the regression figure and a smaller ARE and MSE when compared to the SVM and ER models. The regression results of the GP model for the shear modulus are shown in Figure 5. The MSE of the GP model is 2.23 × 10^−2^ GPa^2^ while that of the ER model is 3.21 × 10^−2^ GPa^2^ and that of the SVM model is 2.89 × 10^−2^ GPa^2^. The R value of the GP model is 0.98136 while that of the ER model is 0.90321 and that of the SVM model is 0.84193. The maximum value of the ARE of the GP model is approximately equal to 35% while that of the ER model is 120% and that of the SVM model is 700%. Therefore, for the shear modulus, the GP model shows the best regression results. We selected it for further performance predictions. 

With a similar system energy, cell volume, Young’s modulus and bulk modulus, the GP models have a better regression performance compared to the ER and SVM models. Specific data can be found in Supplementary Figure S1. We provide the MSE, regression figure and ARE for all models established using the three algorithms in Supplementary Figure S1. Therefore, in this work, we ultimately chose the GP model for subsequent performance prediction work. From Supplemental Figure S1, it can be seen that the machine learning model we have designed does not excessively pursue high R values and low errors, because machine learning is prone to overfitting and underfitting states. Even if all AREs are controlled within 5%, overfitting states may still occur which lead to bad predictions. And when the ARE is above 50%, it is easy to encounter underfitting states. Our goal in this work is to design optimal ML models through parameter design and to apply them to perform predictions, in order to achieve full coverage of property data in all component spaces, rather than just designing machine learning models with minimum error and not applying them to actual situations. Metrics such as the MSE are used as references during model training rather than as targets, so we compare the three algorithms we use, and select one without blindly comparing our MSE values with those of other articles. During the model training process, we also make adjustments based on the predicted results. Finally, we obtain the best GP model. We compare the predictive results from the ML models with the elastic properties of materials reported in other studies to demonstrate the effectiveness of our model. Table 1 displays the validation results for machine learning models in the FeNiCrAlCo system. The relative errors between the results predicted by the machine learning model and the experimental data in the literature are all below 5%, which falls within the range of engineering errors and is acceptable. Other machine learning models also exhibit the same effects. The results fully verify the reliability of the ML models we constructed.

**Figure 6:**
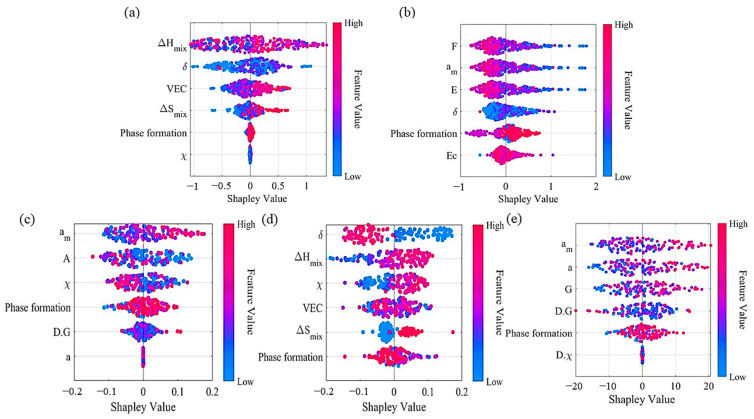
The Shapley values of the GP model for the (**a**) system energy, (**b**) cell volume, (**c**) Young’s modulus, (**d**) bulk modulus and (**e**) shear modulus.

Currently, most ML models lack interpretability, and as black box models we cannot uncover the relationships within them. The required interpretability of the algorithm, just like the reason behind the result, is a problem. So, it is essential to conduct an interpretability analysis of ML models. In this work, we use Shapley values to interpret the relationship between property optimization and the phase and mechanical parameters. This relationship has a certain physical significance, providing the basis for element and component design. The Shapley values of input features can intuitively reflect their contributions to the output [54]. The results of the model’s perturbation explanation are shown in Figure 6. The ordinate of each input feature represents the output quantity with the same Shapley value, and the Shapley values of each input feature are within a limited range. Among them, the most obvious distinction in Shapley values is for the bulk modulus, as shown in Figure 6d. When the difference in atomic radius δ is small, the differences in ΔH_mix_, ΔS_mix_ and χ become larger, leading to greater bulk modulus values. However, the influence of VEC on the bulk modulus is not significant. The phase formation that has a significant impact on the performance exhibits different trends in the moduli E, B and G. When the constituent phase is BCC or FCC, the material is more likely to have a high bulk modulus, while the values of the Young’s modulus and shear modulus are lower. Additionally, the lattice constants a_m_ and a have the same effect on the Young’s modulus and shear modulus. The larger the lattice constants, the higher the modulus values, indicating a better performance. At the same time, when calculating the system energy, the absolute value of the negative numerical value is considered. Figure 6a shows that with an increase in ΔH_mix_ and a decrease in ΔS_mix_ at a fixed temperature, the probability of a larger absolute value of the system energy increases, indicating that the system tends to be more stable. This is consistent with the principles of thermodynamics, as shown in Equation (2).

### 3.3. Multivariate System Prediction

**Figure 7:**
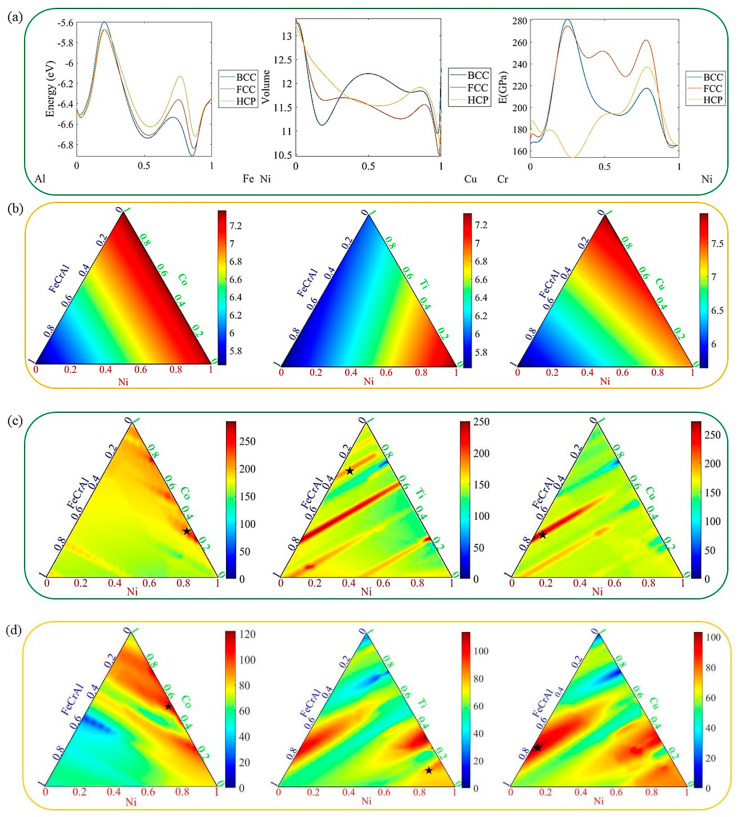
Using the established machine learning model, (**a**) binary phase predictions are made, and using multi-component FeNiCrAlM (M = Co, Cu, Ti) systems as examples to screen out stable structural compositions based on the prediction of (**b**) minimum energy values of phase compositions, in order to obtain the (**c**) Young’s modulus and (**d**) shear modulus of three systems. The stars ‘
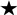
’ represent the single-phase maximum modulus in these alloy systems.

By utilizing machine learning models specifically designed for each output, we achieve predictions of steady-state structures and elastic properties from binary to multi-component systems through spatial transformations. Figure 7a shows some binary prediction results. For energy predictions, the predicted results for the corresponding single-element systems on the left and right of the binary system are consistent with the actual stable structures. In the Ni-Cu system shown here for cell volume predictions, the cell volume of the FCC and HCP dense pack structures is generally smaller than that of the BCC structure, indicating a denser structure, which is consistent with the actual situation and corresponds to a larger density. For the Cr-Ni system, there is an extremum in the Ni content around 0.3 or 0.8.

We take the FeNiCrAlM (M = Co, Cu, Ti) quinary system as an example to demonstrate the predictive performance of the model. Considering the influence of the phase formation on the elastic properties, we use the predicted minimum value of the system energy obtained from the multi-component system to screen out the steady-state structure. Based on this, we predict the elastic properties of the quinary system, as shown in Figure 7c,d. During the prediction process, we find that the predicted result of B is significantly lower than those of E and G, and there is a certain relationship among E, B and G, as shown in Equation (3)

(3) $$B=\frac{EG}{9G-3E}$$

where E (GPa) is the Young’s modulus, B (GPa) is the bulk modulus and G (GPa) is the shear modulus. Therefore, we select E and G for predicting the entire composition space and focus more on optimizing the machine learning model of E and G based on this result. B can be obtained based on E and G. The blue region in Figure 7c,d indicates lower values, while the red region indicates higher values. For the modulus E, when the relative fraction of Co is above 0.5, the material’s Young’s modulus slightly increases, and its tensile strength is also higher. The predicted graphs for Ti and Cu are very similar, with blue regions appearing at composition points of 0.2 and 0.76, corresponding to smaller E values. At 0.32, a higher modulus value is observed. By comparison, it can be seen that Ti has more red regions in the predicted graph compared to Cu. It also exhibits larger modulus values at contents above 0.8 and below 0.24, demonstrating a better strengthening effect. As for the shear modulus, the addition of different components, Co, Cu and Ti, has a significant impact. When the fraction of Co is between 0.30 and 0.45, G is relatively lower, indicating poorer compressive resistance, whereas it is higher in the remaining regions. The addition of Ti with a relative fraction of 0–0.18 slightly increases G, and a maximum value of G is observed for the FeNiCrAlTi system at fractions of 0.25–0.4. When the fraction of Cu is between 0 and 0.20 and 0.28and 0.45, the FeNiCrAlCu system has a higher G, and a minimum value is observed at a fraction of around 0.78. In terms of the modulus G, Co demonstrates a better improvement effect compared to Cu and Ti.

Based on the above work, we conduct an inverse prediction to design multi-component alloys with a high elasticity performance. Our goal is not to design multi-component alloys with the highest elastic modulus, but to design alloys that have higher modulus values than existing ones with the best performance. Taking the five-component alloy system as an example, we can find elements and compositions that have higher elastic moduli. It is noteworthy that the system we select is a single-phase system and we exclude multi-phase alloy systems because the alloy we predict is single-phase alloy. In the predicted results, for modulus E, the Fe_0.01_Cr_0.01_Al_0.01_Ni_0.69_Co_0.28_ alloy with an HCP phase has a modulus of 242.10 GPa, the Fe_0.06_Cr_0.06_Al_0.06_Ni_0.09_Ti_0.73_ alloy with an HCP phase has a modulus of 189.76 GPa, and the Fe_0.23_Cr_0.23_Al_0.23_Ni_0.03_Cu_0.28_ alloy with a BCC phase has the highest modulus value of 273.10 GPa. For modulus G, the Fe_0.01_Cr_0.01_Al_0.01_Ni_0.44_Co_0.53_ alloy with a BCC phase has a modulus of 103.6 GPa, the Fe_0.01_Cr_0.01_Al_0.01_Ni_0.83_Ti_0.14_ alloy with a BCC phase has a modulus of 87.09 GPa, and the Fe_0.25_Cr_0.25_Al_0.25_Ni_0.01_Cu_0.24_ alloy with a BCC phase has a modulus of 102.8 GPa. It can be observed that the alloys selected by us exhibit higher moduli in comparison to the alloys mentioned in Table 1 in other studies. From the above results, it can be seen that most of the modulus maximum values correspond to the BCC phase, which has a better strengthening effect compared to the FCC and HCP. The above results only show the maximum values of the five-component alloy. There may be greater modulus values in some binary and ternary systems, which have better elastic property. The selected component compositions include cases with 0.01 components, which are difficult to accurately obtain in actual preparation processes and should be adjusted based on actual situations.

## 4. Conclusions

This work predicts the elastic properties of the FeNiCrAlCoTiCu alloy in both binary and multi-component systems. By employing input feature selection and parameter design for machine learning models, the risks of overfitting and underfitting, which are typical problems in small dataset machine learning are effectively reduced. We specifically design models for each output that have RMSE values of less than 1.1, and their effectiveness is confirmed through experimental data in the literature. The average relative error between our predicted results and the experimental data in the literature is less than 5%, which meets the requirement for engineering accuracy. We perform interpretable analysis on the ML models, accurately reflecting the changes caused by atomic properties and obtaining thermodynamic verification. This forms a theoretical framework for guiding element and composition design. In the predicted results, we discover composition configurations that exhibit superior elastic properties compared to existing ones. Taking the FeNiCrAlM (M = Co, Cu, Ti) quinary alloy system as an example, we design a single-phase BCC-structure Fe_0.23_Cr_0.23_Al_0.23_Ni_0.03_Cu_0.28_ alloy with a Young’s modulus of 273.10 GPa, as well as a single-phase BCC-structure Fe_0.01_Cr_0.01_Al_0.01_Ni_0.44_Co_0.53_ alloy with a shear modulus of 103.6 GPa. Compared to the alloys that have already been reported above, they exhibit better elastic properties.

We propose a first principles calculation and machine learning-based method for predicting the steady-state structure and elastic properties of multi-component alloys. This includes a first principles calculation process that considers the phase composition and has a gradient component space, as well as a machine learning model training that accelerates calculations without sacrificing accuracy. Using our research way, we achieve a fast improvement in the calculation speed of first principles calculation for steady-state structure and elastic property predictions, covering the entire composition space of multi-component alloys. Our research way provides a general computing paradigm to accelerate first principles calculations. Our interpretable ML models provide a fast and full-coverage prediction of the elastic properties of multi-component alloys with the accuracy of DFT calculations. By changing the initial element types and data, our research way can be used for a variety of multi-component alloy performance prediction problems.

Our research way only uses the calculation results of unit and binary systems. By transforming the input space, we have achieved rapid property prediction for multi-component systems, resulting in a significant reduction in the cost of first principles calculations. Based on the predicted results, reverse prediction can be performed to screen for elements and composition ratios with higher elastic moduli, thus helping accelerate the design of multi-component alloy materials.

## Figures and Tables

**Table 1:** Comparison of prediction results of the optimal model with the literature.

No.	Element Mass Fraction/wt					E/GPa		B/GPa		G/GPa		Literature
	Fe	Ni	Cr	Al	Co	EV	PV	EV	PV	EV	PV	
1	0.25	0.25	0.25	-	0.25	214	209.86	147	140.87	86	90.55	[52]
2	0.20	0.20	0.20	0.20	0.20	230	233.11	154.13	149.69	94.56	93.32	[52]
3	0.25	0.25	0.23	0.02	0.25	203	199.78	-	-	-	-	[53]
4	0.29	0.11	0.26	0.07	0.27	235	232.76	-		-	-	[53]
5	0.12	0.29	0.04	0.40	0.15	187	185.49	-	-	-	-	[53]

Note: EV—experiment value in the literature; PV—mean predictive value.

## Data Availability

All data generated or analyzed in this study are included in this manuscript and Supplementary Materials.

## Supplementary Materials

The following supporting information can be downloaded at: https://www.mdpi.com/article/10.3390/ma16186226/s1, Table S1: Features for the phase parametric candidates of machine learning models; Table S2. Features for the mechanical parametric candidates of machine learning models; Table S3. The specifically designed parameters of Gaussian Processes models; Figure S1. The (a) MSE, (b) regression figures and (c) ARE of five outputs ML models trained with ER, SVM and GP algorithms.

## References

[1] Zhang W., Liaw P.K., Zhang Y. (2018). Science and technology in high-entropy alloys. Sci. China Mater..

[2] Yeh J.W., Chen S.K., Lin S.J., Gan J.Y., Chin T.S., Shun T.T., Tsau C.H., Chang S.Y. (2004). Nanostructured high-entropy alloys with multiple principal element: Novel alloy design concept and outcomes. Adv. Eng. Mater..

[3] Zhang Y., Yang X., Liaw P.K. (2012). Alloy design and properties optimization of high-entropy alloys. J. Miner. Met. Mater. Soc..

[4] Li M., Zhang Q., Han B., Song L., Li J., Yang J. (2020). Investigation on microstructure and properties of AlxCoCrFeMnNi high entropy alloys by ultrasonic impact treatment. J. Alloys Compd..

[5] Zhang Y., Zuo T.T., Tang Z., Gao M.C., Dahmen K.A., Liaw P.K., Lu Z.P. (2014). Microstructures and properties of high-entropy alloys. Prog. Mater. Sci..

[6] Lu W., Luo X., Yang Y., Le W., Huang B., Li P. (2020). Co-free non-equilibrium medium-entropy alloy with outstanding tensile properties. J. Alloys Compd..

[7] Han Y., Li H., Feng H., Li K., Tian Y., Jiang Z. (2020). Enhancing the strength and ductility of CoCrFeMnNi high-entropy alloy by nitrogen addition. Mater. Sci. Eng..

[8] Thiel F., Geissler D., Nielsch K., Kauffmann A., Seils S., Heilmaier M., Utt D., Albe K., Motylenko M., Rafaja D. (2020). Origins of strength and plasticity in the precious metal based high-entropy alloy AuCuNiPdPt. Acta Mater..

[9] Průša F., Cabibbo M., Šenková A., Kučera V., Veselka Z., Školáková A., Vojtěch D., Cibulková J., Čapek J. (2020). High-strength ultrafine-grained CoCrFeNiNb high-entropy alloy prepared by mechanical alloying: Properties and strengthening mechanism. J. Alloys Compd..

[10] Tian F., Varga L.K., Chen N., Shen J., Vitos L. (2015). Empirical design of single phase high-entropy alloys with high hardness. Intermetallics.

[11] Yang S., Liu Z., Pi J. (2020). Microstructure and wear behavior of the AlCrFeCoNi high-entropy alloy fabricated by additive manufacturing. Mater. Lett..

[12] Soni V., Gwalani B., Alam T., Dasari S., Zheng Y., Senkov O.N., Miracle D., Banerjee R. (2020). Phase inversion in a two-phase, BCC+B2, refractory high entropy alloy. Acta Mater..

[13] Zhao Y.L., Yang T., Li Y.R., Fan L., Han B., Jiao Z.B., Chen D., Liu C.T., Kai J.J. (2020). Superior high-temperature properties and deformation-induced planar faults in a novel L12-strengthened high-entropy alloy. Acta Mater..

[14] Ye Y.F., Wang Q., Lu J., Liu C.T., Yang Y. (2016). High-entropy alloy: Challenges and prospects. Mater. Today.

[15] Qiao L., Zhu J., Teng Y., Bao A., Lai Z., Wang Y. (2021). Dynamic solidification model of low-density FeCrNiAl multi-component alloy. Vacuum.

[16] Yang D., Liu Y., Jiang H., Liao M., Qu N., Han T., Lai Z., Zhu J. (2020). A novel FeCrNiAlTi-based high entropy alloy strengthened by refined grains. J. Alloys Compd..

[17] Jumaev E., Hong S.H., Kim J.T., Park H.J., Kim Y.S., Mun S.C., Park J.-Y., Song G., Lee J.K., Min B.H. (2019). Chemical evolution-induced strengthening on AlCoCrNi dual-phase high-entropy alloy with high specific strength. J. Alloys Compd..

[18] Kalidindi S.R., Brough D.B., Li S., Cecen A., Blekh A.L., Congo F.Y.P., Campbell C. (2016). Role of materials data science and informatics in accelerated materials innovation. MRS Bull..

[19] Brunton S.L., Kutz J.N. (2019). Methods for data-driven multiscale model discovery for materials. J. Phys. Mater..

[20] Hohenberg P., Kohn W. (1964). Inhomogeneous electron gas. Phys. Rev..

[21] Mooney C.Z. (1997). Monte Carlo Simulation.

[22] Alder B.J., Wainwright T.E. (1959). Studies in molecular dynamics. I. General method. J. Chem. Phys..

[23] Boettinger W.J., Warren J.A., Beckermann C., Karma A. (2002). Phase-field simulation of solidification. Annu. Rev. Mater. Res..

[24] Strang G., Fix G.J., Griffin D.S. (1974). An Analysis of the Finite-Element Method.

[25] Saal J.E., Berglund I.S., Sebastian J.T., Liaw P.K., Olson G.B. (2018). Equilibrium high entropy alloy phase stability from experiments and thermodynamic modeling. Scr. Mater..

[26] Ma D., Yao M., Pradeep K.G., Tasan C.C., Springer H., Raabe D. (2015). Phase stability of non-equiatomic CoCrFeMnNi high entropy alloys. Acta. Mater..

[27] Choi W.-M., Jo Y.H., Sohn S.S., Lee S., Lee B.-J. (2018). Understanding the physical metallurgy of the CoCrFeMnNi high-entropy alloy: An atomistic simulation study. NPJ Comput. Mater..

[28] Jarlöv A., Ji W., Zhu Z., Tian Y., Babicheva R., An R., Seet H.L., Nai M.L.S., Zhou K. (2022). Molecular dynamics study on the strengthening mechanisms of Cr-Fe-Co-Ni high-entropy alloys based on the generalized stacking fault energy. J. Alloys Compd..

[29] Ma D., Grabowski B., Körmann F., Neugebauer J., Raabe D. (2015). Ab initio thermodynamics of the CoCrFeMnNi high entropy alloy: Importance of entropy contributions beyond the configurational one. Acta. Mater..

[30] Lederer Y., Toher C., Vecchio K.S., Curtarolo S. (2018). The search for high entropy alloys: A high-throughput ab-initio approach. Acta Mater..

[31] Jo Y.H., Choi W.M., Kim D.G., Zargaran A., Sohn S.S., Kim H.S., Lee B.J., Kim N.J., Lee S. (2019). FCC to BCC transformation-induced plasticity based on thermodynamic phase stability in novel V10Cr10Fe45CoxNi35−x medium-entropy alloys. Sci. Rep..

[32] Schleder G.R., Padilha A.C.M., Acosta C.M., Costa M., Fazzio A. (2019). From DFT to machine learning: Recent approaches to materials science-a review. J. Phys. Mater..

[33] Zhang L., Qian K., Huang J., Liu M., Shibuta Y. (2021). Molecular dynamics simulation and machine learning of mechanical response in non-equiatomic FeCrNiCoMn high-entropy alloy. J. Mater. Res. Technol..

[34] Dey S., Sultana N., Kaiser M.S., Dey P., Datta S. (2016). Computational intelligence based design of age-hardenable aluminium alloys for different temperature regimes. Mater. Des..

[35] Suh J.S., Suh B.-C., Lee S.E., Bae J.H., Moon B.G. (2022). Quantitative analysis of mechanical properties associated with aging treatment and microstructure in Mg-Al-Zn alloys through machine learning. J. Mater. Sci. Technol..

[36] Chen Y., Tian Y., Zhou Y., Fang D., Ding X., Sun J., Xue D. (2020). Machine learning assisted multi-objective optimization for materials processing parameters: A case study in Mg alloy. J. Alloys Compd..

[37] Peng J., Yamamoto Y., Hawk J.A., Lara-Curzio E., Shin D. (2020). Coupling physics in machine learning to predict properties of high-temperatures alloys. NPJ Comput. Mater..

[38] Du J.L., Feng Y.L., Zhang M. (2021). Construction of a machine-learning-based prediction model for mechanical properties of ultra-fine-grained Fe-C alloy. J. Mater. Res. Technol..

[39] Guo T., Wu L., Li T. (2021). Machine learning accelerated, high throughput, multi-objective optimization of multiprincipal element alloys. Small.

[40] Guo T., Li J., Wang J., Wang W.Y., Liu Y., Luo X., Kou H., Beaugnon E. (2018). Microstructure and properties of bulk Al0.5CoCrFeNi high-entropy alloy by cold rolling and subsequent annealing. Mater. Sci. Eng..

[41] Fu Z., Chen W., Xiao H., Zhou L., Zhu D., Yang S. (2013). Fabrication and properties of nanocrystalline Co0.5FeNiCrTi0.5 high entropy alloy by MA-SPS technique. Mater. Des..

[42] Nam S., Kim M.J., Hwang J.Y., Choi H. (2018). Strengthening of Al0.15CoCrCuFeNiTi–C (x = 0, 1, 2) high-entropy alloys by grain refinement and using nanoscale carbides via powder metallurgical route. J. Alloys Comp..

[43] Zhang A., Han J., Meng J., Su B., Li P. (2016). Rapid preparation of AlCoCrFeNi high entropy alloy by spark plasma sintering from elemental powder mixture. Mater. Lett..

[44] Roy A., Taufique M.F.N., Khakurel H., Devanathan R., Johnson D.D., Balasubramanian G. (2022). Machine-learning-guided descriptor selection for predicting corrosion resistance in multi-principal element alloys. NPJ Mater. Degrad..

[45] Zhang L., Chen H., Tao X., Cai H., Liu J., Ouyang Y., Peng Q., Du Y. (2020). Machine learning reveals the importance of the formation enthalpy and atom-size difference in forming phases of high entropy alloys. Mater. Des..

[46] Zhang Y., Zhou J., Lin P., Chen L., Liaw K. (2008). Solid-Solution Phase Formation Rules for Multi-component Alloys. Adv. Eng. Mater..

[47] Li X., Wu S., Li X., Yuan H., Zhao D. (2020). Particle Swarm Optimization-Support Vector Machine Model for Machinery Fault Diagnoses in High-Voltage Circuit Breakers. Chin. J. Mech. Eng..

[48] Guo Y., Wang X., Xiao P., Xu X. (2020). An ensemble learning framework for convolutional neural network based on multiple classifiers. Soft Comput..

[49] Deringer V.L., Bartók A.P., Bernstein N., Wilkins D.M., Ceriotti M., Csányi G. (2021). Gaussian Process Regression for Materials and Molecules. Chem. Rev..

[50] Choudhury A. (2021). The Role of Machine Learning Algorithms in Materials Science: A State of Art Review on Industry 4.0. Arch. Comput. Methods Eng..

[51] Hu Y.J., Zhao G., Zhang B., Yang C., Zhang M., Liu Z.K., Qian X., Qi L. (2019). Local electronic descriptors for solute-defect interactions in bcc refractory metals. Nat. Commun..

[52] Koval E.N., Juaristi J.I., Muiño R.D., Alducin M. (2020). Structure and properties of CoCrFeNiX multi-principal element alloys from ab initio calculations. J. Appl. Phys..

[53] Yang T., Xia S., Liu S., Wang C., Liu S., Zhang Y., Xue J., Yan S., Wang Y. (2015). Effects of AL addition on microstructure and mechanical properties of Al CoCrFeNi High-entropy alloy. Mater. Sci. Eng. A.

[54] Yang J., Cao J. (2021). Tree-based interpretable machine learning of the thermodynamic phases. Phys. Lett. A.

