# Supplementary material for: Accelerating First Principles Calculation of Multi-Component Alloy Steady-State Structure and Elastic Properties in Full Component Space
Source: Materials (Basel). 2023 Sep 15;16(18):6226. doi: 10.3390/ma16186226 (PMC10532447; doi:10.3390/ma16186226)
Supplement: Supplementary file 1 [file materials-16-06226-s001.zip › materials-2604498-supplementary.pdf]

Table S1. Features for the phase parametric candidates of machine learning models.

Parameter	Calculation Formula	Description	Parameter	Calculation Formula	Description
δ	$\sqrt{\sum_{i=1}^{n} C_i(1-\frac{r_i}{r})^2}$	Differences in Atomic Radii	T_m	$\sum_{i=1}^{n} C_i T_i$	The melting point calculated by the mixing law
χ	$\sqrt{\sum_{i=1}^{n} C_i(\chi_i-\bar{\chi})^2}$	Electronegativity difference	a_m	$\sum_{i=1}^{n} C_i a_i$	The lattice constant calculated by the mixing rule
VEC	$\sum_{i=1}^{n} C_i VEC_i$	Valence electron concentration	ΔT_m	$\sqrt{\sum_{i=1}^{n} C_i(T_i-\bar{T})^2}$	Melting point difference
ΔH_{mix}	$\sum_{i=1,i\neq j}^{n} 4H_{ij}C_i C_j$	Mixed Flame	a	$\sqrt{\sum_{i=1}^{n} C_i(a_i-\bar{a})^2}$	Melting point difference
ΔS_{mix}	$-R\sum_{i=1}^{n}(C_i \ln C_i)$	Mixed Xiu	ρ	$\sum_{i=1}^{n} C_i \rho_i$	Melting point difference
Λ	$\Delta S_{mix/(\delta * \delta}$	Λ parameter	δ_{con}	$\sqrt{\sum_{i=1}^{n} C_i(1-\frac{r_{coni}}{r_{con}})^2}$	Covalent radius difference
γ	$(1-\sqrt{\frac{(r+r_{min})^2-r^2}{(r+r_{min})^2}})/(1-\frac{(r+r_{miax})^2-r^2}{(r+r_{max})^2})$	γ parameter	I	$\sum_{i=1}^{n} C_i I_i$	Ionization energy

D. χ	$\sum_{i=1}^{n}\sum_{j=1,i\neq j}^{n} C_iC_j * \lvert\chi_i-\chi_j\rvert$	Local electronegativity mismatch	A_r	$\sum_{i=1}^{n} C_iA_{ri}$	Relative atomic mass
E_c	$\sum_{i=1}^{n} C_i * (Ec)_i$	Cohesion	EA	$\sum_{i=1}^{n} C_iEA_i$	Electronic affinity work
λ	$\frac{\Delta S_{mix}}{\delta^2}$	Geometric parameters			

Table S2. Features for the mechanical parametric candidates of machine learning models.

Parameter	Calculation Formula	Description	Parameter	Calculation Formula	Description
η	$\sum_{i=1}^{n}\frac{C_i * \frac{2(G_i-G)}{G_i+G}}{1+0.5*\left\lvert C_i * \frac{2(G_i-G)}{G_i+G}\right\rvert}$	The modulus in the reinforcement model does not match	δG	$\sqrt{\sum_{i=1}^{n} C_i * (1-\frac{G_i}{G})^2}$	Difference in shear modulus
D. r	$\sum_{i=1}^{n}\sum_{j=1,i\neq j}^{n} C_iC_j * \lvert r_i-r_j\rvert$	Partial size mismatch	D. G	$\sum_{i=1}^{n}\sum_{j=1,i\neq j}^{n} C_iC_j * \lvert G_i-G_j\rvert$	Local modulus mismatch
A	$G * \delta r * (1+\mu)(1-\mu)$	Strengthen the energy term in the model	G	$\sum_{i=1}^{n} C_i * G_i$	Shear modulus

F	$\frac{2G}{1-\mu}$	Peierls-Nabarro factor	E	$\sum_{i=1}^{n} C_i * E_i$	Young’s modulus
μ	$\frac{1}{2}E * \delta$	Lattice distortion energy	υ	$\frac{E_i}{2G_i} - 1$	Poisson's ratio

Table S3. The specifically designed parameters of Gaussian Processes models.

Output	Parameters
System Energy	'Crossval', 'on'; 'Fitmethod', 'exact'; 'ComputationMethod', 'qr'; 'Sigma', 0.025; 'Regularization', 0.1; 'PredictMethod', 'exact'
Cell Volume	'Crossval', 'on'; 'Fitmethod', 'exact'; 'ComputationMethod', 'qr'; 'Sigma', 0.025; 'Regularization', 0.01; 'PredictMethod', 'exact'
Young’s Modulus	'Crossval', 'on'; 'Holdout', 0.3; 'Fitmethod', 'exact'; 'ComputationMethod', 'qr'; 'Standardize', true; 'BasisFunction', 'linear'; 'KernelFunction', 'ardsquaredexponential'; 'PredictMethod', 'exact'; 'ConstantSigma', true; 'Sigma', 0.02; 'Regularization', 0.06; 'BasisFunction', 'constant'
Bulk Modulus	'Crossval', 'on'; 'Holdout', 0.2; 'Fitmethod', 'sr'; 'ComputationMethod', 'qr'; 'Standardize', true; 'KernelFunction', 'ardsquaredexponential'; 'PredictMethod', 'sr'; 'ConstantSigma', true; 'Sigma', 0.003; 'BasisFunction', 'linear'
Shear Modulus	'Crossval', 'on'; 'Fitmethod', 'none'; 'KernelFunction', 'exponential'; 'BasisFunction', 'none'; 'ComputationMethod', 'v'; 'ConstantSigma', false; 'Regularization', 0.01; 'PredictMethod', 'sr'

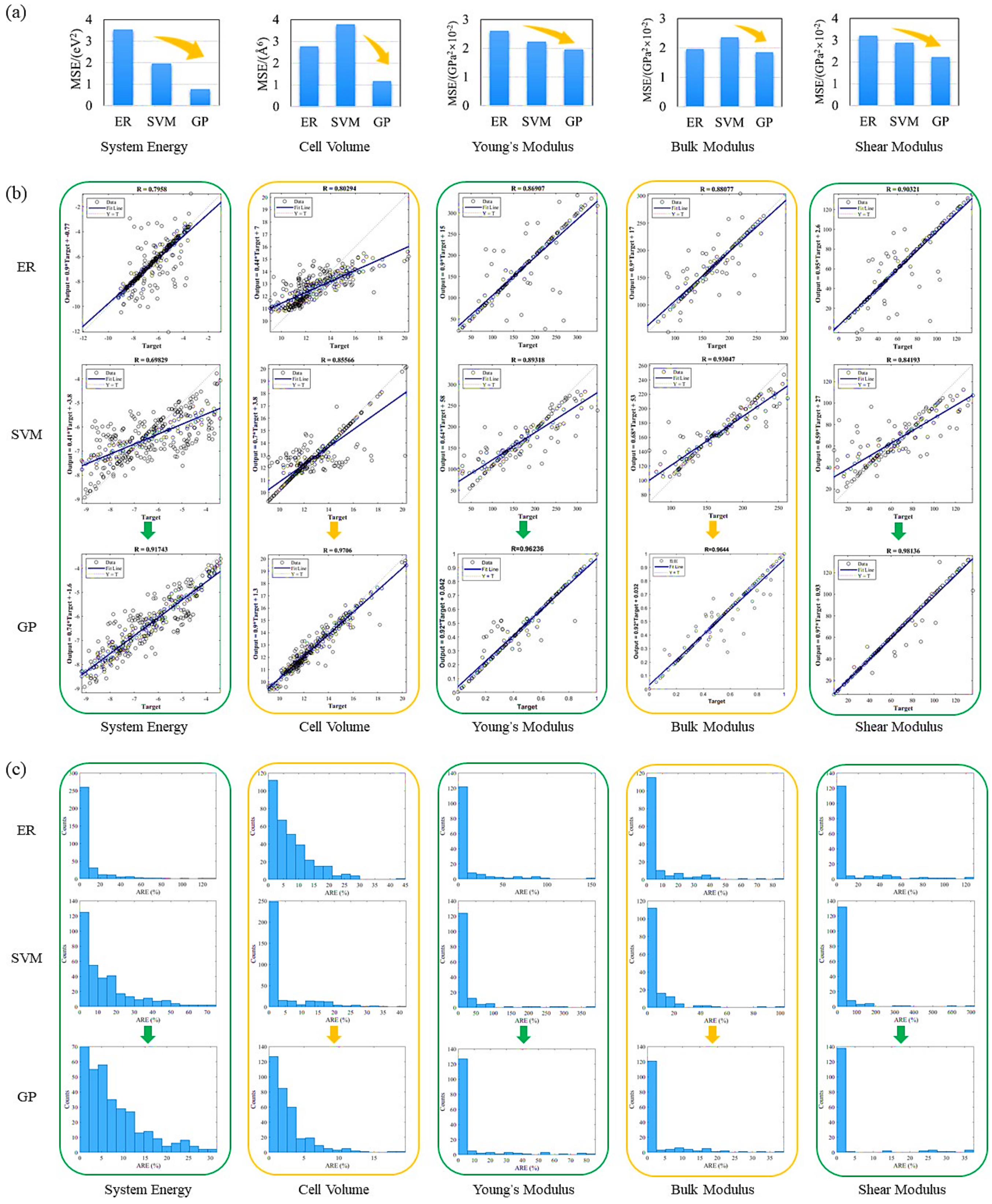

Figure S1. The (a) MSE, (b) regression figures and (c) ARE of five outputs ML models trained with ER, SVM and GP algorithms.
